# Glucocorticoids Promote Extracellular Matrix Component Remodeling by Activating YAP in Human Retinal Capillary Endothelial Cells

**DOI:** 10.3389/fcell.2021.738341

**Published:** 2021-12-14

**Authors:** Xianliang Gu, Lingling Ge, Bangqi Ren, Yajie Fang, Yijian Li, Yi Wang, Haiwei Xu

**Affiliations:** ^1^ Southwest Hospital/Southwest Eye Hospital, Third Military Medical University (Army Medical University), Chongqing, China; ^2^ Key Lab of Visual Damage & Regeneration and Restoration of Chongqing, Chongqing, China

**Keywords:** glucocorticoids, diabetic retinopathy, endothelial cells, YAP, extracellular matrix

## Abstract

Remodeling of extracellular matrix (ECM) components of endothelial cells is the main cause of retinal vascular basement membrane (BM) thickening, which leads to the initiation and perpetuation of microvasculopathy of diabetic retinopathy (DR). Excessive amounts of glucocorticoids (GCs) are related to the presence and severity of DR, however transcriptional effects of GCs on the biology of human retinal capillary endothelial cells (HRCECs) and its impacts on DR are still unclear. Here, we showed that GC (hydrocortisone) treatment induced ECM component [fibronectin (FN) and type IV collagen (Col IV)] expression and morphological changes in HRCECs via the glucocorticoid receptor (GR), which depended on the nuclear translocation of YAP coactivator. Mechanistically, GCs induced stress fiber formation in HRCECs, while blocking stress fiber formation inhibited GC-induced YAP nuclear translocation. Overexpression of FN, but not Col IV, activated YAP through the promotion of stress fiber formation via ECM-integrin signaling. Thus, a feedforward loop is established to sustain YAP activity. Using mRNA sequencing of HRCECs with overexpressed YAP or GC treatment, we found a similarity in Gene Ontology (GO) terms, differentially expressed genes (DEGs) and transcription factors (TFs) between the two RNA-seq datasets. *In vivo*, YAP was activated in retina vascular ECs of STZ-induced diabetic mice, and TF prediction analysis of published RNA-seq data of dermal vascular ECs from T2DM patients showed that GR and TEAD (the main transcription factor for YAP) were enriched. Together, GCs activate YAP and promote ECM component (FN and Col IV) remodeling in retinal capillary endothelial cells, and the underlying regulatory mechanism may provide new insights into the vascular BM thickening of the retina in the early pathogenesis of DR.

## Introduction

Diabetic retinopathy (DR) is a sight-threatening pathological condition that affects almost 100 million people worldwide and is influenced by many risk factors (Leasher et al., 2016; Stitt et al., 2016). Despite years of research and numerous clinical trials, the etiology and pathology of DR remain unclear (Stitt et al., 2016). Appropriate control of systemic risk factors, such as hyperglycemia, hypertension, and dyslipidemia, postpones the development of this disease (Nathan et al., 1993; Turner, 1998; Yau et al., 2012; Chew et al., 2014). However, all three factors account for less than 10% of the DR risk (Hirsch and Brownlee, 2010). This finding suggests that additional unidentified factors may also participate in the initiation and perpetuation of DR.

The classification and grading of DR is based on the severity of microvascular lesions, such as microaneurysms, hemorrhages, venous beading and neovascularization, since these pathologies are obvious during disease progression (Stitt et al., 2016; Flaxel et al., 2020). The cellular pathologies of vascular lesions include blood-retinal barrier (BRB) dysfunction, basement membrane (BM) thickening, and pericyte and capillary endothelial cell (EC) death (Stitt et al., 2016). ECs, which line the inner lumenal surfaces of retinal blood vessels, are covered by pericytes or vascular smooth muscle cells and participate in many homoeostatic processes (Yu et al., 2014). In the retinas, ECs are an important part of the inner BRB, and are the main cell type that secretes BM components (Roy et al., 2010; Yu et al., 2014). In DR, capillary EC dysfunction is known to be the primary defect, and the BM thickening is considered one of the main causes of cell dysfunction (Stitt et al., 2016). This BM alteration may contribute to impaired EC-pericyte communication, defects in the selective permeability barrier or inappropriate interactions of cells with constitutive BM proteins, all of which lead to apoptosis (of ECs, pericytes, etc.) (Stitt et al., 2016). Animal experiments have shown that preventing early BM thickening of retinal capillaries can attenuate early vascular changes caused by diabetes mellitus (Van Geest et al., 2014), indicating that EC production of excess BM components is a key pathway of initiation and progression of microvasculopathy in DR, but the mechanism remains to be elucidated.

Some clinical trials have reported that the presence and severity of DR in subjects with type 2 diabetes mellitus (T2DM) is related to the degree of secretion of cortisol (Oltmanns et al., 2006; Chiodini et al., 2007), which is the only endogenous glucocorticoid (GC) in humans (Cain and Cidlowski, 2015). Metabolic syndrome, which is strongly associated with aberrant GC signaling, has also been found to be associated with T2DM-related microvascular disease (Vegiopoulos and Herzig, 2007; Lee et al., 2018). Such data strongly indicate that hypercortisolism may be a risk factor of DR. Chronic hypercortisolism, even if mild, has major clinical consequences, including increased risks for metabolic diseases (glucose, lipid and bone metabolism-related diseases) and cardiovascular diseases (CVDs), compromised immune responses and increased mortality (De Leo et al., 2010; Del Rincón et al., 2014; Geer, 2016). Naturally, hypercortisolism might contribute to worsening of DR by causing metabolic disorder. In addition, because GCs can directly interact with cells of the heart and vascular wall during the genesis and development of CVDs (Hadoke et al., 2009; Liu et al., 2019), we speculate that excess amounts of cortisol may directly worsen DR by regulating transcription and protein synthesis in retinal cells.

ECs are important targets for GCs, which can exert both positive and negative effects. In inflammation-related retinal edema, intravitreal injection of exogenous GCs can maintain endothelial barrier integrity (Zielińska et al., 2016); however, it is also associated with angiostatic effects, including reductions in choroidal and retinal vascular density (Valamanesh et al., 2009). Endogenous GCs can induce some physiological changes, including vascular tone and arterial blood pressure changes (Goodwin et al., 2011), and respond to some CVDs, including atherosclerosis, hypertension, and central serous chorioretinopathy (CSC) (Daruich et al., 2015b; Liu et al., 2019). In both of the pathological and physiological changes of the cardiovascular system, endothelial GR has been shown to be intimately involved, indicating that GCs can directly affect ECs. Mechanically, GCs can promote proliferation or apoptosis, expression of anti- or proinflammatory cytokines, and expression of junctional and extracellular matrix (ECM) proteins of ECs, depending on the different physiological and pathological conditions and EC subtype (Weidenfeller et al., 2005; Blecharz et al., 2008; El Zaoui et al., 2015; Thomsen et al., 2017). In DR, GCs may induce retinal capillary EC dysfunction through the above molecular mechanism.

Yes-associated protein (YAP) is a transcription cofactor, which controls multiple aspects of cell behavior, including proliferation, migration and cell plasticity (Totaro et al., 2018). Recent studies have revealed that YAP plays a critical role in sprouting angiogenesis and vascular barrier maturation by regulating the migration, proliferation and expression of junctional and ECM proteins of ECs in the retina and brain (Kim et al., 2017). This molecule is also critically involved in pathological angiogenesis, such as choroidal neovascularization (Yan et al., 2018; Feng et al., 2021). In addition, GCs have been proven to be new upstream signals for YAP (Sorrentino et al., 2017). Therefore, YAP could be a potential effector molecule in GC-induced EC dysfunction.

Despite the accumulating and compelling data supporting the roles of GCs in the pathogeneses of some vascular diseases via specific ECs (Liu et al., 2019), few studies have investigated the direct effects of GCs on human retinal capillary EC (HRCEC) biology and the relevant impacts on DR. Here, to fill this knowledge gap, we explored the responsiveness of HRCECs to GCs from the perspectives of cell morphology, extracellular matrix (ECM) component expression, gene expression profiles, and cell functions *in vitro*. We showed that GCs promote the expression of fibronectin (FN) and type IV collagen (Col IV) by activating the transcriptional coactivator YAP in HRCECs. Mechanistically, using immunofluorescence, transcriptomic analysis, pharmacological treatments, and overexpression of YAP with cell cultures, we found that GC treatment increased YAP nuclear accumulation via the glucocorticoid receptor (GR), and YAP-mediated FN overexpression led to ECM-integrin pathway stimulation and stress fiber-dependent YAP activation. In turn, YAP increases FN expression, indicating the formation of a positive feedback response. Using a diabetic mouse model and published RNA-seq data of T2DM patients, we confirmed that YAP and GR were activated in vascular ECs *in vivo*. In summary, GCs are novel regulators of ECM component expression in HRCECs, and the underlying regulatory mechanism provides new insights into the understanding of the vascular BM thickening of the retina in DR.

## Materials and Methods

### Reagents, Plasmids, and Lentiviruses

Hydrocortisone (IH0100, Solarbio), RU486 (Mifepristone, M8046, Sigma Aldrich), verteporfin (VTP, HY-B0146, MedChemExpress), mitomycin C (Mc, S8146, Selleck), latrunculin A (lat.A, ab144290, Abcam), Arg-Gly-Asp (RGD, 52305, GL Biochem (Shanghai) Ltd.), fluorescein-phalloidin (P5282, Sigma; A12380, Invitrogen), human plasma fibronectin (FN) (F0895, Sigma), and collagen type IV (Col IV) from human cell culture (C6745, Sigma). All the aforementioned compounds were prepared into stock solutions according to the manufacturers’ instructions.

pQCXIH-Myc-YAP-5SA was a gift from Kunliang Guan (Addgene plasmid #33093; http://n2t.net/addgene:33093; RRID: Addgene_33093). pQCXIH was from YouBia (VT1535, Hunan, China).

Lentiviral constructs coding for human Yes-associated protein 1 (YAP1) (clone ID: NM_001130145) (Lentiviral-YAP1-EGFP) and a control lentivirus (GV358 empty vector) with EGFP expression were obtained from GeneChem (Shanghai, China).

### Antibodies

Several antibodies were used for immunofluorescence, FACS and western blotting (WB). The primary antibodies included a mouse anti-FN monoclonal antibody (ab6328, Abcam; 1:400), a rabbit anti-FN polyclonal antibody (ab2413, Abcam; 1:500), a rabbit anti-Col IV alpha 1 polyclonal antibody (ab189408, Abcam; 1:500), a rabbit anti-YAP1 polyclonal antibody (ab39361, Abcam; 1:400), a rabbit anti-YAP1 (phospho-S127) monoclonal antibody (ab76252, Abcam; 1:3000), a rabbit anti-CD31 polyclonal antibody (ab182981, Abcam; 1:500), a monoclonal anti-Ki-67-APC antibody (130-100-330, Miltenyi; 1:11), a monoclonal REA-APC control (I) antibody (130-104-615, Miltenyi; 1:11), and a mouse anti-GAPDH monoclonal antibody (CW0100M, CWBIO; 1:3000). The secondary antibodies included goat anti-rabbit Alexa Fluor-568 (A11011, Life Technologies; 1:500), goat anti-rabbit Alexa Fluor-488 (A11001, Life Technologies; 1:400), goat anti-mouse Alexa Fluor-488 (A32723, Life Technologies; 1:400), goat anti-mouse Alexa Fluor-568 (A11031, Life Technologies; 1:500), and horseradish peroxidase (HRP)-conjugated antibodies (goat anti-rabbit/mouse; A0208, A0216, Beyotime; 1:3000).

### Cell Cultures and Morphological Observation

HRCECs were obtained from Guangzhou Jennio Biotech Co., Ltd. (Guangzhou, China). The culture medium for this cell line was RPMI 1640 medium (Gibco) containing 10% fetal bovine serum (FBS; Gibco). The HRCEC cultures were maintained at 37.0°C in a humidified atmosphere containing 5% CO_2_. The groups described below were all cultured in 6-well plates (1.5 × 10^4^ cells/well), and the morphology was observed at 72 h under a DM IRE2 light microscope (Leica). HRCECs cultured in RPMI 1640 medium (Gibco) containing 10% FBS (Gibco) for 72 h were termed the normally treated (NT) group. HRCECs cultured in RPMI 1640 medium (Gibco) containing 10% FBS (Gibco) and 0.8 μM hydrocortisone for 72 h were termed the GC group.

### Retinal Sections, Cell Climbing Slide Preparation and Immunofluorescence Staining

A diabetes mellitus (DM) mouse model was established as previously described (Rong et al., 2018). The C57BL/6 mice used in the present study were housed according to the Third Military Medical University (Army Medical University) guidelines. The animal protocols were conducted according to the relevant guidelines approved by the Laboratory Animal Welfare and Ethics Committee of Third Military Medical University (Army Medical University). After the mice were sacrificed, their eyeballs were prefixed in 4% paraformaldehyde (PFA) at room temperature for 30 min, and then, the anterior segments were removed under a microscope (Olympus) and fixed in 4% PFA for another 2 h. After dehydration in 30% sucrose overnight at 4°C, the eye cups were embedded in Tissue-Tek OCT compound, and then 15-mm-thick sections were cut using a Leica cryostat and attached to glass slides. HRCECs were seeded on cell slides at a density of 2 × 10^4^ cells/cm^2^ and fixed in 4% PFA at 4°C for 15 min when they reached 80% confluence.

For immunofluorescence staining, retinal sections and cells on cell climbing slides were permeabilized in 0.1% Triton X-100 for 10 min and then blocked in 5% goat serum for 60 min. Next, the slides were incubated with the indicated primary antibodies at 4°C overnight and subsequently with fluorophore-conjugated secondary antibodies at 37°C for 1 h after 3 rinses with phosphate-buffered saline (PBS). After counterstaining with DAPI, all slides were immersed in antifade medium and mounted on glass slides. All samples were viewed and photographed using a confocal microscope (Leica SP5/8; Zeiss LSM800).

### RNA Extraction and Real-Time Quantitative PCR

Total RNA was extracted from the retinas of DM mice 3 months after induction or from HRCECs after the indicated treatments using RNAiso Plus (Takara) as described by the manufacturer. After the quality of RNA was ensured with a NanoDrop 2000 spectrophotometer (Thermo Scientific), the RNA was reverse-transcribed into cDNA using a PrimeScript™ RT Reagent Kit with gDNA Eraser (Takara). Real-time qPCR was performed on a CFX96 Real-Time System (Bio-Rad) using cDNA, SYBR Green Premix Ex Taq (Takara) and gene-specific primers. The relative expression levels of the genes of interest were normalized to the expression of GAPDH and were calculated using the delta-delta Ct method. The primers used in this study are shown in Supplementary Table 1.

### Flow Cytometry

For analysis of cell proliferation, HRCECs that had been cultured with GC or not for 72 h were dissociated using trypsin (HyClone) and then pretreated with a Fixation/Permeabilization Kit (554714, BD Biosciences) according to the manufacturer’s instructions. Anti-Ki67 and corresponding isotype control antibodies were added to the fixed cell suspensions, which were then incubated in the dark for 30 min at 4°C. After they were washed and resuspended in Perm/Wash™ Buffer (554714, BD Biosciences), the stained cells were detected using flow cytometry (FACSCalibur, BD Biosciences) and analyzed with FlowJo X 10.0.7r2 software (TreeStar, Inc.).

An apoptosis assay was performed using an Apoptosis Detection Kit (556547, BD Biosciences). Briefly, cells were detached from plates with TrypLE Express (Gibco) and incubated in Annexin V (AV) binding buffer with propidium iodide (PI) and APC-AV at room temperature for 15 min in the dark. Without washing and resuspension, the stained cells were assayed using flow cytometry within 1 h.

HRCECs transfected with Lentiviral-YAP1-EGFP were dissociated into single-cell suspensions and loaded into a flow cytometer (MoFlo XDP, Beckman Coulter). EGFP-labeled and unlabeled cells were collected and stored in RNAiso Plus (Takara) at −80°C.

### *In Vitro* Scratch Wound Healing Assay

HRCECs were plated onto 6-well plates, each with a horizontal line drawn on the back, and treated with different conditioned media. When the HRCECs had grown into a confluent monolayer, three vertical scratches were made across the center of each well using a sterile 200 μl pipette tip. After three washes with PBS to remove any floating cells, the HRCECs were cultured in serum-free RPMI 1640 supplemented with the indicated reagents. The intersections of the horizontal line with the vertical scratches were the observed positions. Using a DM IRE2 light microscope (Leica), phase-contrast images of the wound were obtained at the observed positions at 0, 12, and 24 h. The scratch area was quantified using ImageJ software. The percentage of wound closure was determined as the healed area at each time point/initial scratch area.

### Enzyme-Linked Immunosorbent Assay

The culture media from HRCECs after the indicated treatments were collected and stored at −80 C for double-antibody sandwich ELISA. According to the manufacturer’s instructions, the concentration of FN in culture supernatants were measured with a FN ELISA kit (SEA037Hu, Cloud-Clone Crop, China) and the concentration of Col IV in culture supernatants were measured with a Col IV ELISA kit (SEA180Hu, Cloud-Clone Crop, China). The relative ECM components (FN and Col IV) secretion levels of HRCECs after the indicated treatments were normalized to the secretion level of NT or GC group.

### WB

The cell pellets were lysed in an ice-cold mixture of RIPA buffer with PMSF (Beyotime, Shanghai, China), and total protein was extracted from the lysed cells after centrifugation in a refrigerated centrifuge. The protein concentration was measured using the BCA assay method (Beyotime). After denaturation in loading buffer at 100°C for 10 min, equal amounts of protein were loaded onto 8–20% SDS-PAGE gels for electrophoresis and electrophoretically transferred onto methanol-activated polyvinylidene difluoride (PVDF) membranes (0.45 mm; Millipore). The membranes were blocked with 5% BSA dissolved in TBST (20 mM Tris, 145 mM NaCl (pH 7.6) and 0.02% Tween 20) for 2 h at room temperature and then incubated with primary antibodies at 4°C overnight. The following day, the membranes were washed with TBST (three times for 15 min) and incubated with proper secondary HRP-conjugated antibodies for 2 h at room temperature. The immunoreactive bands were detected by reacting them with enhanced chemiluminescence (ECL) reagents (Pierce) under a ChemiDoc MP Imaging System (Bio-Rad).

### Plasmid Transfection and Lentivirus Infection

HRCECs were seeded in 6-well plates and were deemed ready for transfection when they had reached 80% confluence. For plasmid transfection, Lipofectamine 3000 (Thermo Scientific) and the plasmid pQCXIH-Myc-YAP-5SA were added concomitantly to the dish and incubated for 72 h, as instructed; the plasmid pQCXIH was used as a control. For lentivirus infection, HRCECs were infected with a YAP1 overexpression (YAP1-OE) or control lentivirus at a multiplicity of infection (MOI) of 30. The infectious medium (RPMI 1640 with 2% FBS and 4% infectious reagent) was replaced with fresh medium after 12 h. Culture was performed for an additional 60 h for subsequent FACS or RNA analyses.

### Immunofluorescence Image Analysis

Stress fibers were defined as fiber-like structures of rearranged filamentous actin (F-actin) oriented in the longitudinal direction of the cell and crossing the nucleus and were identified using phalloidin staining. The numbers of stress fiber-positive cells and nuclei labeled by DAPI were counted in the same photomicrographs, and the corresponding ratios were calculated.

To analyze YAP subcellular localization, cellular borders were identified with phalloidin staining (indicated by the most peripheral phalloidin signals), and nuclear localization was determined using a DAPI mask. YAP staining intensity in the nucleus and cytoplasm was measured, and the ratio between nuclear and cytoplasmic YAP (N/C ratio) was calculated.

Nuclear area measurement was performed with isolated nuclei from cells that were not in the dividing phase. After manually selecting nuclei with the Magic Wand Tool, the areas were calculated with the measuring tool in Photoshop (Adobe Systems).

Integrated fluorescence intensity of individual cells was measured using FIJI.

### RNA-Seq and Bioinformatics Analysis

HRCECs transfected with Lentiviral-YAP1-EGFP were termed the YAP-OE group. HRCECs transfected with the GV358 empty vector were termed the control group. The two RNA-seq tests (GC vs. NT and control vs. YAP-OE) were performed according to the same instructions. Briefly, total RNA of samples was extracted using TRIzol reagent (Invitrogen), and mRNA was enriched with oligo (dT) magnetic beads and fragmented into short fragments using fragmentation buffer. cDNA libraries were constructed by PCR amplification of the fragments using an Agilent 2100 Bioanalyzer and an ABI StepOnePlus Real-Time PCR System. The qualified libraries were sequenced through the Illumina HiSeq platform with a 150-bp paired-end model, and clean reads were obtained after qualification and filtration. The fragments per kilobase of transcript per million fragments mapped (FPKM) values were calculated for differentially expressed gene (DEG) analysis between the two groups using the DEGseq package. Genes with adjusted *p* values ≤ 0.05 were considered to be DEGs.

Gene Ontology (GO) term enrichment analysis and correlation analysis among listed genes or significantly enriched GO terms were performed using Metascape (http://metascape.org/). The “princomp” function in RStudio (R version 1.1.463) was used for principal component analysis (PCA), and the ggplot 2 package in R was used for graph drawing.

RNA-seq data of dermal ECs of T2DM patients were retrieved from GSE92724 (Wimmer et al., 2019). We used the DEGseq package to calculate the DEGs with adjusted *p* values ≤ 0.05. Enrichment analysis was conducted using Metascape (http://metascape.org/).

The Mining Algorithm for GenetIc Controllers (MAGIC) and a web-accessible software system called oPOSSUM-3 (http://opossum.cisreg.ca) were used to predict transcription factors (TFs) using the DEGs. The TF binding sites with corresponding Z-scores and Fisher scores > the means of their respective distributions were considered enriched in oPOSSUM-3 analysis, and TFs with corrected *p* values < 10% were considered enriched in MAGIC analysis.

A raincloud plot and Venn diagram were generated in the bioinformatics platform (http://www.bioinformatics.com.cn).

### FN and Col IV Treatment

HRCECs were seeded into 6-well plates coated with 6.25 μg/cm^2^ FN according to the manufacturer’s instructions. Briefly, a 0.1% (5 mg) FN solution was diluted to 60 μg/ml in sterile water, and 1 ml of the FN solution was added to one well of each 6-well plate and allowed to coat the well for 1 h at room temperature. This experimental group was termed the FN (6.25) group, and the group to which RGD was added at a final concentration of 1 mg/ml was termed the FN (6.25)+RGD group. In addition, HRCECs were seeded into 6-well plates coated with 2.0, 4.0, and 8.0 μg/cm^2^ Col IV. Col IV solution (300 μg/ml) was diluted to 19.2, 38.4 and 76.8 μg/ml, and 1 ml of each dilution solution was added to one well of each 6-well plate. Next, the plates were incubated at 37°C for 1 h. The experimental groups were termed the Col IV (2.0), Col IV (4.0), and Col IV (8.0) groups, respectively.

### Transmission Electron Microscopy

Retinas derived from DM mice 3 months after induction and control mice were fixed in 2.5% glutaric dialdehyde/0.1 M cacodylate buffer for 16 h and subsequently postfixed in 1% osmium tetroxide for 2 h. After dehydration with an ascending acetone series and soaking in acetone/resin (1:1) for 4 h, the retinas were embedded in epoxy resin 618. Ultrathin sections (1 μm) were counterstained with lead and 3% uranyl acetate and observed under a JEM-1400 Plus electron microscope (JEOL Inc.). The basement membrane thickness of retinal capillaries was measured using Photoshop.

### Statistical Analysis

Statistical calculations were performed using SPSS version 18.0 (IBM, Inc.) and Prism 6 software (GraphPad). All quantitative variables are presented as the mean ± standard deviation, except variables in the raincloud plot (Figure 4E), for which the Tukey method was used to plot the whiskers and outliers. Unpaired or paired two-tailed Student’s t-test and one-way ANOVA with Dunnett’s test were used for pairwise comparisons and multigroup comparisons, respectively. Statistical analysis of intersections in Venn diagrams was performed by hypergeometric tests (one-tailed Fisher’s exact test). *p* values < 0.05 were considered significant.

## Results

### GC Treatment Induces ECM Component Expression and Morphological Changes in HRCECs Via GR

**FIGURE 1:**
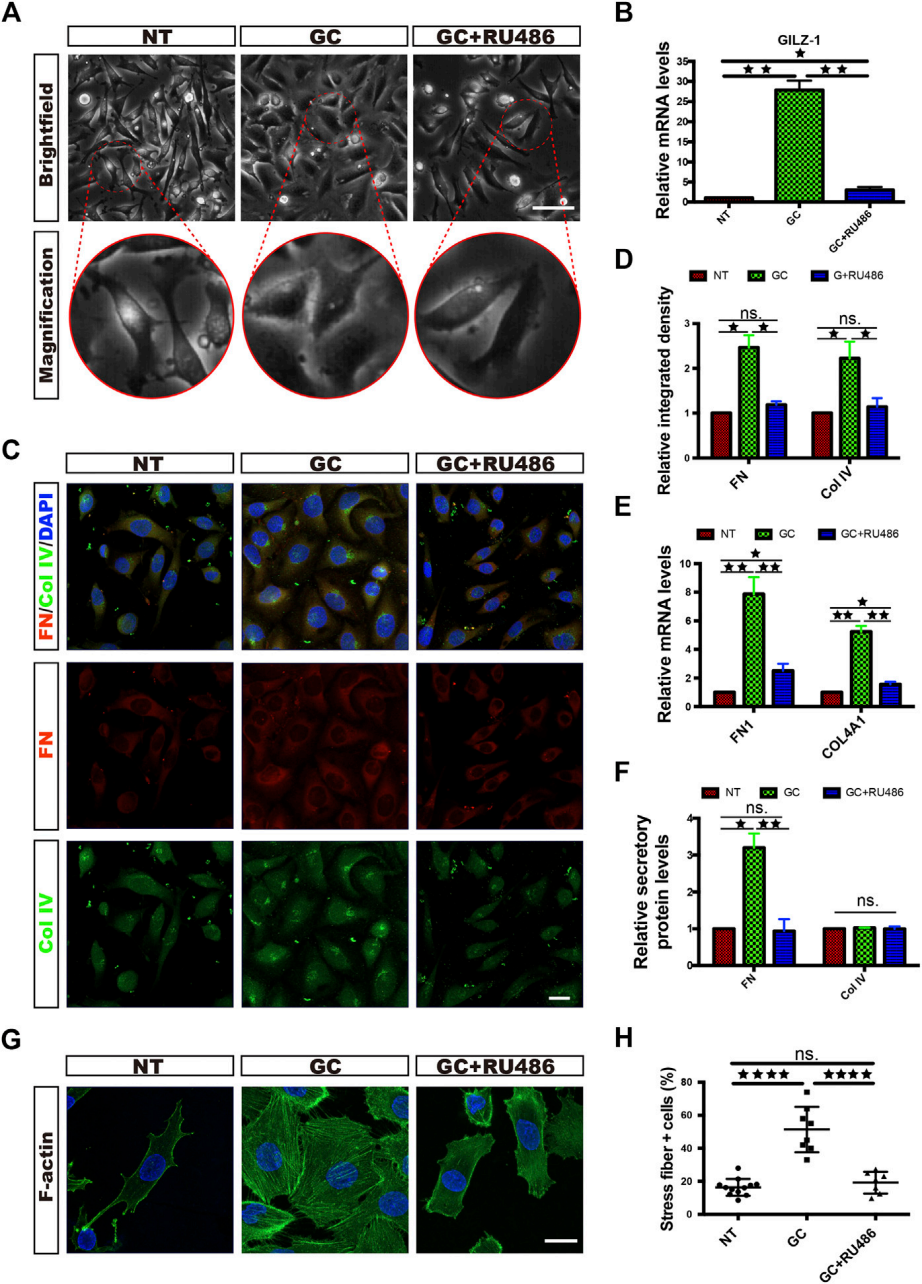
GCs induce ECM component expression and morphological changes in HRCECs via a GC receptor. **(A)**: Brightfield microscopy images of HRCECs treated with 0.8 μM hydrocortisone (GC) alone or in combination with 10 μM RU486 (GC + RU486) for 72 h. The NT HRCECs were cultured in RPMI 1640 medium (Gibco) containing 10% FBS (Gibco). Scale bars, 100 μm. **(B)**: Real-time qPCR analysis of GILZ in HRCECs treated as in **(A)**. **(C)**: FN/Col IV/DAPI immunofluorescence staining of HRCECs treated as in **(A)**. Scale bars, 20 μm. **(D)**: Quantification of FN and Col IV integrated intensity of individual HRCECs treated as in **(A)**. **(E)**: Real-time qPCR analysis of FN1 and COL4A1 in HRCECs treated as in **(A)**. **(F)**: FN and Col IV secretion in supernatant of HRCECs treated as in **(A)** as analyzed via ELISA followed by normalization to NT group. **(G)**: F-actin was stained by immunofluorescence in HRCECs treated as in **(A)**. Scale bars, 20 μm. **(H)**: Quantification of the percentage of cells displaying actin stress fibers for the three treatment conditions in **(A)**. The error bars represent the mean ± SD, and the experiment was biologically repeated three times. *p < 0.05; **p < 0.01; ****p < 0.0001; ns., not significant. Student’s two-tailed paired t-test **(B,D,E,F)** or Student’s two-tailed unpaired t-test **(H)**. ECM extracellular matrix, FN fibronectin, Col IV type IV collagen.

To explore the responsiveness of HRCECs to GCs, we cultured HRCECs with a GC (hydrocortisone), and we found that the HRCECs underwent marked morphological changes with clear increases in cell size and spreading and exhibited cell edges that were blurred instead of sharp. All of the changes were prevented by concomitant GC and RU486 (GR receptor inhibitor) administration (Figure 1A). The light microscopy analysis indicated that GCs can induce ECM component expression and morphological changes in HRCECs via GR. Semiquantitative analysis of the immunofluorescence of FN and Col IV and real time-qPCR analysis of FN1 and COL4A1 suggested that GCs can induce ECM component expression (Figures 1C,E). Moreover, the secretion level of FN was significantly increased under GC treatment (Figure 1F). Immunofluorescence staining of F-actin and corresponding cell counting showed that GC treatment led to the formation of prominent actin stress fibers extending across the cell body in response to an elongated cell morphology (Figures 1G,H). Changes in the mRNA expression of GILZ, the target gene of GR, showed that RU486 blocked the abovementioned changes in HRCECs induced by GC treatment, as determined by immunofluorescence staining, real-time qPCR, cell counting and ELISA (Figures 1B,D–F,H).

**FIGURE 2:**
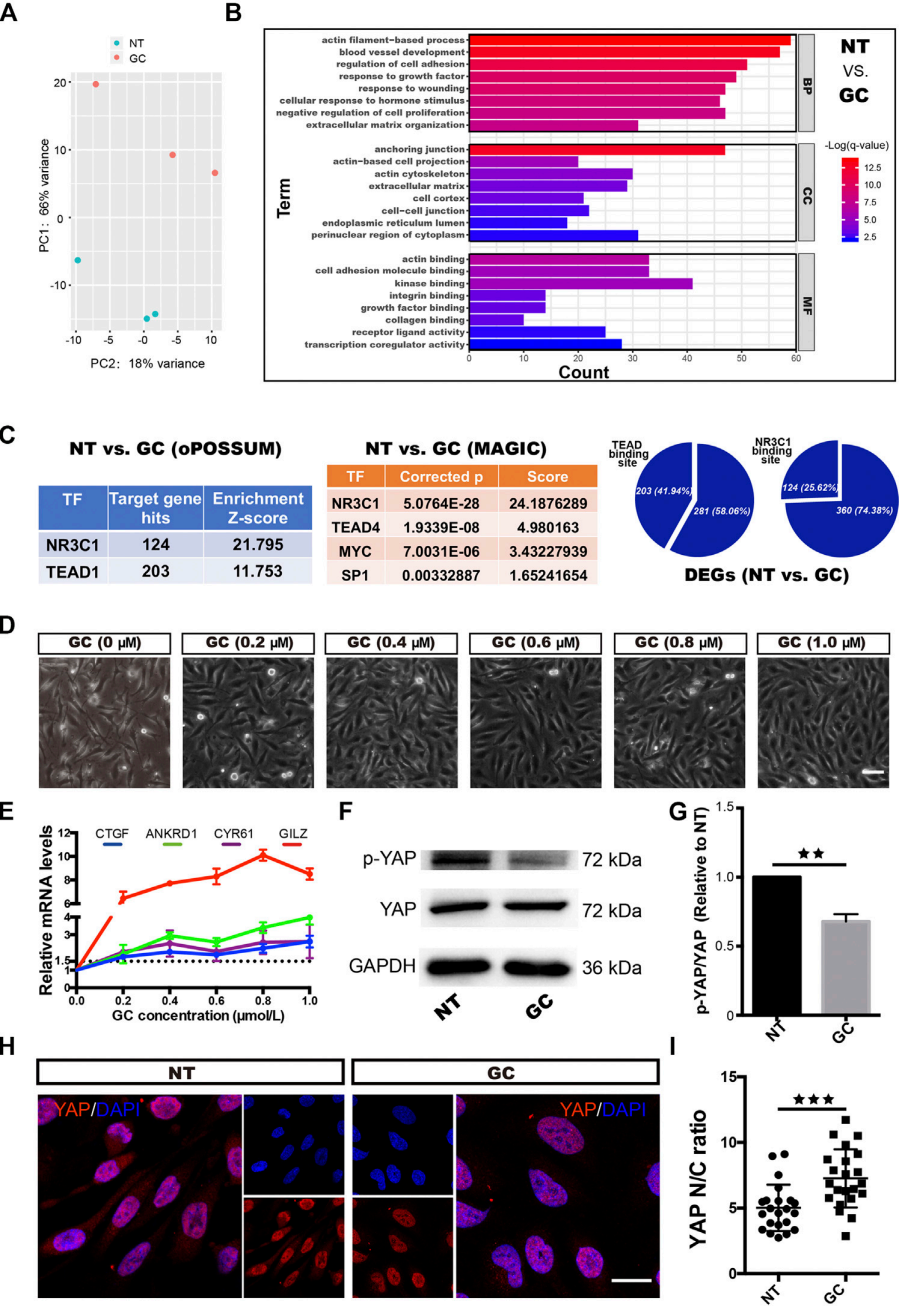
GCs induce YAP activation in HRCECs. **(A)**: PCA was performed on samples from the NT group and the GC group. HRCECs were cultured in RPMI 1640 medium (Gibco) containing 10% FBS (Gibco) alone (NT group) or in combination with 0.8 μM hydrocortisone (GC group) for 72 h. **(B)**: Top significantly enriched GO terms for the DEGs between the NT group and the GC group for the BP, MF and CC categories. **(C)**: Selected factors found in the TF binding site enrichment analysis of DEGs from the NT vs. GC comparisons. The analysis was performed through two methods. oPOSSUM-3 is a web-accessible software program. The MAGIC algorithm was used for analysis in Python. Proportion of significantly differentially regulated genes in the DEGs with predicted TEAD and NR3C1 binding sites in promoter regions. **(D)**: Brightfield microscopy image of HRCECs treated with different concentrations of hydrocortisone (GC) for 72 h. Scale bars, 100 μm. **(E)**: Real-time qPCR analysis of HRCECs treated as in **(D)**. mRNA levels of GILZ (a target of GR) and ANKRD1/CYR61/CTGF (targets of YAP) are calculated. **(F):** WB analysis of YAP and p-YAP of HRCECs treated with or without 0.8 μM hydrocortisone for 72 h p-YAP is phospho-S127. **(G)**: The p-YAP/YAP in **(F)** is quantified and normalized to NT group. **(H)**: YAP was stained by immunofluorescence in HRCECs treated as in **(F)**. Scale bars, 20 μm. **(I)**: Quantification of the ratio of the average immunofluorescence intensity of YAP between the nucleus and the cytoplasm of the cells in **(H)**. The error bars represent the mean ± SD, and the experiment was biologically repeated three times. **p < 0.01, ***p < 0.001. Student’s two-tailed paired t-test **(G)** or Student’s two-tailed unpaired t-test **(I)**. DEGs differentially expressed genes, GO Gene Ontology, BP biological process, CC cellular component, MF molecular function, TF transcription factor.

With increasing GC concentrations, the changes in HRCECs became increasingly obvious (Figure 2D) and real-time qPCR showed that the expression of GILZ increased gradually, peaking under 0.8 μM treatment (Figure 2E); thus, a concentration of 0.8 μM GC was used for the subsequent experiments.

In addition, we found that the GC promoted the proliferation and migration of HRCECs but did not affect apoptosis (Supplementary Figure 1).

### GCs Induce ECM Expression and Morphological Changes in HRCECs Depending on YAP Nuclear Localization

We next sought to elucidate the mechanism of the dramatic changes in HRCECs (the results shown in Figure 1 and Supplementary Figure 1) under GC treatment. We conducted an RNA-seq analysis of the NT and GC groups. PCA showed that samples from the NT group could be separated from the GC group samples (Figure 2A). The top significantly enriched GO terms for the DEGs between the NT group and the GC group in the biological process (BP), molecular function (MF) and cellular component (CC) categories were examined, and terms such as “actin filament-based process”, “extracellular matrix organization”, and “cellular response to hormone stimulus” were consistent with the previous results *in vitro* (Figure 2B). To dissect the global transcriptional networks in the HRCECs treated with GC, we used two methods to perform a TF prediction analysis and found that not only NR3C1 (the name of the gene encoding GR) but also TEAD (transcriptional enhancer factor TEF with TEA/ATTS domain) was enriched in the RNA-seq dataset (Figure 2C). Moreover, the number of genes with predicted TEAD-binding sites in their promoter regions was far more than those with predicted NR3C1-binding sites [203 (41.94%) vs. 124 (25.62%)] (Figure 2C). TEAD transcription factors cannot activate transcription unless they interact with the nuclear coactivators, such as YAP (Gibault et al., 2018). YAP shuttles between the cytoplasm and the nucleus and mainly regulates downstream gene expression by interacting with TEAD family members, such as TEAD1–4 (Yagi et al., 1999; Stein et al., 2015). Overrepresented conserved transcription factor binding sites in the DEGs included sites for AP1, MYC, and SP1 (Figure 2C), which have been reported to cooperate with YAP-TEAD, supporting the existence of the YAP-TEAD interaction in our study. We previously found the GC treatment induces morphological changes, proliferation and migration of HRCECs, and combined with the analysis of RNA-seq data, they suggest that YAP-TEAD may mediate these changes in HRCECs.

As GC concentrations increased, the mRNA levels of CYR61, ANKRD1 and CTGF, three downstream transcriptional target genes of YAP, were also increased (Figure 2E). Immunofluorescence staining revealed that more cells displayed an increased percentage of nuclear-localized YAP in the GC group than in the NT group (Figure 2H). Moreover, quantification of the ratio of the average YAP immunofluorescence intensity between the nucleus and the cytoplasm confirmed that GCs can induce YAP nuclear localization (Figure 2I). Alongside YAP nuclear localization, GCs inhibited S127 phosphorylation on YAP but did not affect the total YAP protein level (Figures 2F,G).

**FIGURE 3:**
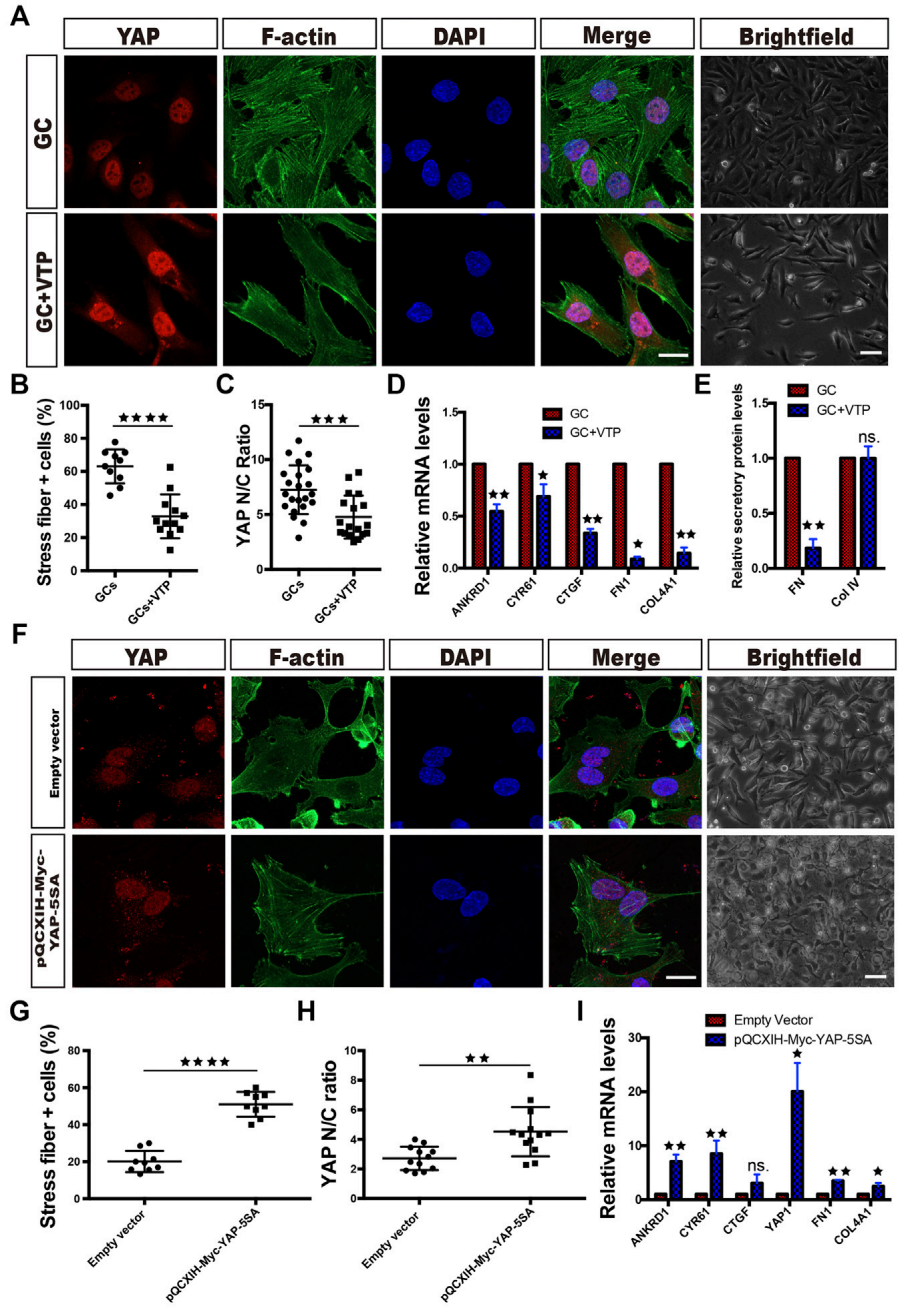
YAP activity controls ECM component expression and morphological changes in HRCECs. **(A)**: YAP/F-actin/DAPI immunofluorescence staining of HRCECs treated with 0.8 μM hydrocortisone alone (GC) or in combination with 10 μM VTP (GC + VTP) for 72 h. Scale bars, 20 μm. Brightfield microscopy images of HRCECs treated in the indicated condition. Scale bars, 100 μm. **(B)**: Quantification of the percentage of cells displaying actin stress fibers for the two treatment conditions in **(A)**. **(C)**: Quantification of the ratio of the average immunofluorescence intensity of YAP between the nucleus and the cytoplasm of the cells in **(A)**. **(D)**: Real-time qPCR analysis of HRCECs treated as in **(A)**. The mRNA levels of FN1/COL4A1 (ECM component) and ANKRD1/CYR61/CTGF (targets of YAP) were calculated. The Real-time qPCR analysis of ANKRD1/CYR61/CTGF was biologically repeated five times, and the analysis of FN1/COL4A1 was biologically repeated three times. **(E)**: FN and Col IV secretion in supernatant of HRCECs treated as in **(A)** as analyzed via ELISA followed by normalization to GC group. **(F)**: YAP/F-actin/DAPI immunofluorescence staining of HRCECs transduced with the YAP-5SA plasmid or not. Scale bars, 20 μm. Brightfield microscopy images of HRCECs treated under the indicated condition. Scale bars, 100 μm. **(G)**: Quantification of the percentage of cells displaying actin stress fibers for the two treatment conditions in **(F)**. **(H)**: Quantification of the ratio of the average immunofluorescence intensity of YAP between the nucleus and the cytoplasm of the cells in **(F)**. **(I)**: Real-time qPCR analysis of HRCECs treated as in **(F)**. The mRNA levels of FN1/COL4A1 (ECM component), YAP1 and ANKRD1/CYR61/CTGF (targets of YAP) were calculated. The error bars represent the mean ± SD, and all experiments were biologically repeated three times, except the experiment in **(D)**. *p < 0.05; **p < 0.01; ***p < 0.001, ****p < 0.0001; ns., not significant. Student’s two-tailed paired t-test **(D,E,I)** or Student’s two-tailed unpaired t-test **(B,C,G,H)**. ECM extracellular matrix, VTP verteporfin.

When VTP, a pharmacological inhibitor of YAP that disrupts YAP-TEAD signaling, was administered concomitantly with the GCs, retention of YAP in the cytoplasm increased (Figures 3A,C). The results also showed that VTP reversed the ECM component (FN and Col IV) expression, FN secretion, stress fiber formation and morphological changes that were induced by GC treatment (Figures 3A,B,D,E).

We also transfected HRCECs with the plasmid pQCXIH-Myc-YAP-5SA, which encodes active YAP. The cells transfected with pQCXIH-Myc-YAP-5SA had significantly higher YAP1 mRNA expression than the cells supplied with an empty vector and showed overexpression of nuclear YAP along with stress fiber formation (Figures 3F–I). These cells also expressed more FN1 and Col4A1 mRNA than empty vector-transfected cells (Figure 3I).

### Blocking Stress Fiber Formation Inhibits GC-Induced YAP Nuclear Translocation

YAP activity is mainly regulated by the conformation and tension of the F-actin cytoskeleton (Totaro et al., 2018). In the RNA-Seq analysis of the NT and GC groups, many GO terms with the word “actin” were the most significantly enriched, such as the actin filament-based process, actin-based cell projection, actin cytoskeleton and actin binding terms (Figure 2B). Combined with the result that GCs induce stress fiber formation in HRCECs (Figures 1G,H), these findings indicate that stress fiber formation plays a vital role in the regulation of YAP by GCs.

**FIGURE 4:**
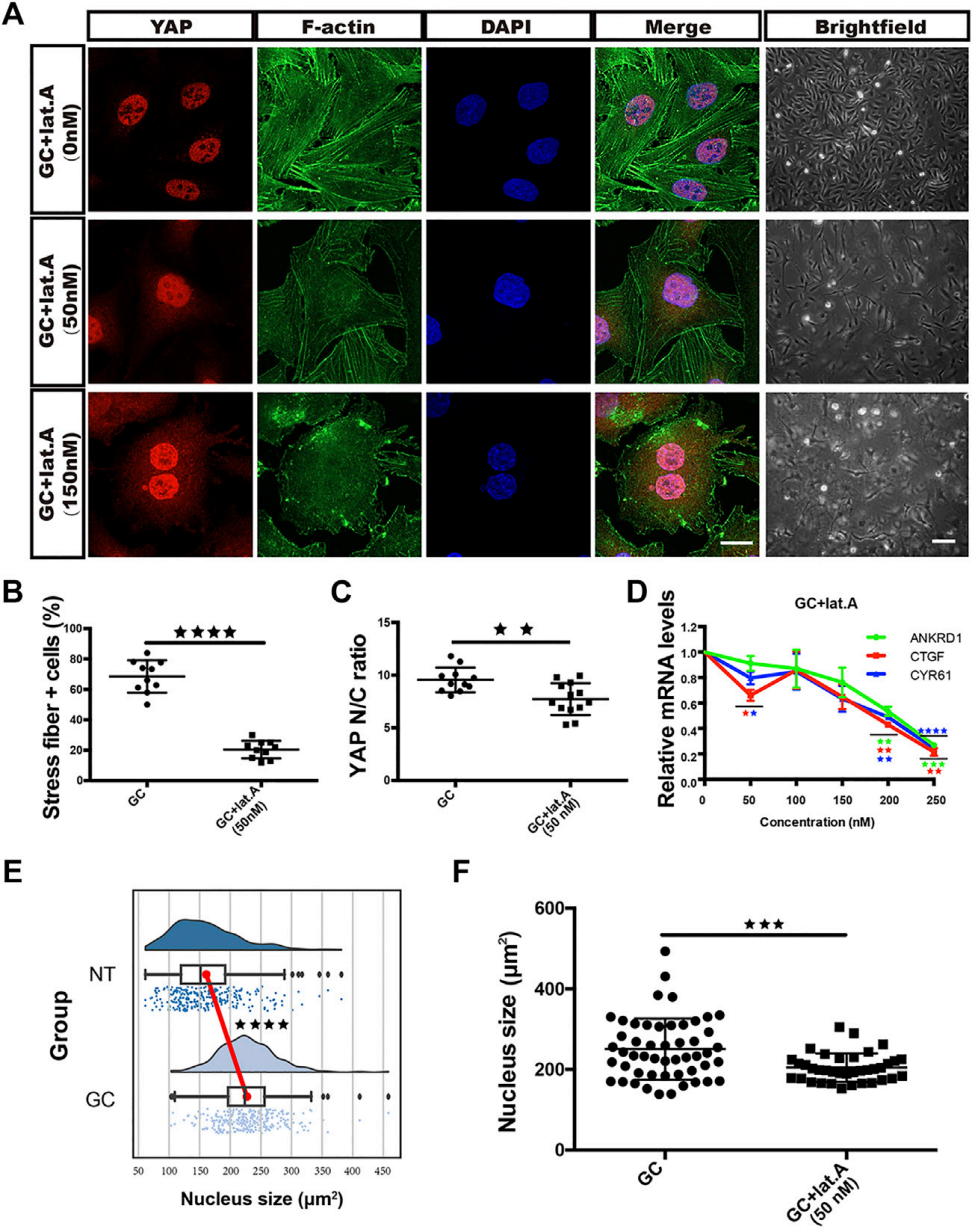
GCs induce YAP nuclear translocation via stress fiber formation-dependent flattening of the nucleus. **(A)**: YAP/F-actin/DAPI immunofluorescence staining of HRCECs treated with 0.8 μM hydrocortisone alone or in combination with 50 nM or 150 nM lat.A for 72 h. Scale bars, 20 μm. Brightfield microscopy images of HRCECs treated under the indicated condition. Scale bars, 200 μm. **(B)**: Quantification of the percentage of cells displaying actin stress fibers for the three treatment conditions in **(A)**. **(C)**: Quantification of the ratio of the average immunofluorescence intensity of YAP between the nucleus and the cytoplasm of the cells in **(A)**. **(D)**: Real-time qPCR analysis of HRCECs treated with 0.8 μM hydrocortisone alone or in combination with 50 nM, 100 nM, 150 nM, 200 nM or 250 nM lat.A for 72 h. The mRNA levels of ANKRD1/CYR61/CTGF (targets of YAP) were calculated. **(E)**: Quantification of the nucleus size of HRCECs form NT and GC group via immunofluorescence images. **(F)**: Quantification of the nucleus size of HRCECs treated as in **(A)** from immunofluorescence images. The error bars represent the mean ± SD **(B,C,D,F)**. The Tukey method was used to plot the whiskers and outliers **(E)**, and the experiment was biologically repeated three times. **p < 0.01; ***p < 0.001; ***p < 0.0001. Student’s two-tailed unpaired t-test **(B,C,E,F)** or one-way ANOVA followed by Dunnett’s multiple comparison tests **(D)**. lat.A latrunculin A.

We pharmacologically inhibited actin polymerization in GC-treated cells with lat.A. Strikingly, as the lat.A concentration increased, the proportion of cells with stress fiber formation and the YAP nuclear entry induced by GC treatment gradually decreased (Figures 4A–C). In addition, the GC-induced morphological changes and changes in the expression of YAP target genes (CYR61, ANKRD1 and CTGF) were all prevented by lat.A (Figures 4A,D).

Roca-Cusachs et al. reported that stress fiber formation-mediated force transmission can lead to nuclear flattening, which stretches nuclear pores, reduces their mechanical resistance to molecular transport, and increases YAP nuclear import (Elosegui-Artola et al., 2017). Whether the nucleus is flattened can be calculated by measuring the area of the nucleus in an immunofluorescence image. We calculated the nuclear areas of cells from the NT and GC groups and found that GC treatment promoted nuclear enlargement (Figure 4E), while lat.A reversed this change (Figure 4F).

### FN Partially Promotes Stress Fiber Formation, YAP Nuclear Translocation and Morphological Changes in HRCECs Via ECM-Integrin Signaling

Mechanotransduction involves the transmission of mechanical signals emanating from the cell microenvironment into cell-specific transcriptional programs (Vogel and Sheetz, 2006). YAP is a well-known mechanotransducer (Panciera et al., 2017). Cells respond to the topology and rigidity of the ECM, which are major extracellular mechanical cues, by adjusting their F-actin structures through integrin-mediated cell-ECM interactions, and the eventual effect is YAP activation-mediated gene expression to modulate cell differentiation, proliferation, apoptosis and migration (Aragona et al., 2013; Gaspar and Tapon, 2014; Mohri et al., 2017).

**FIGURE 5:**
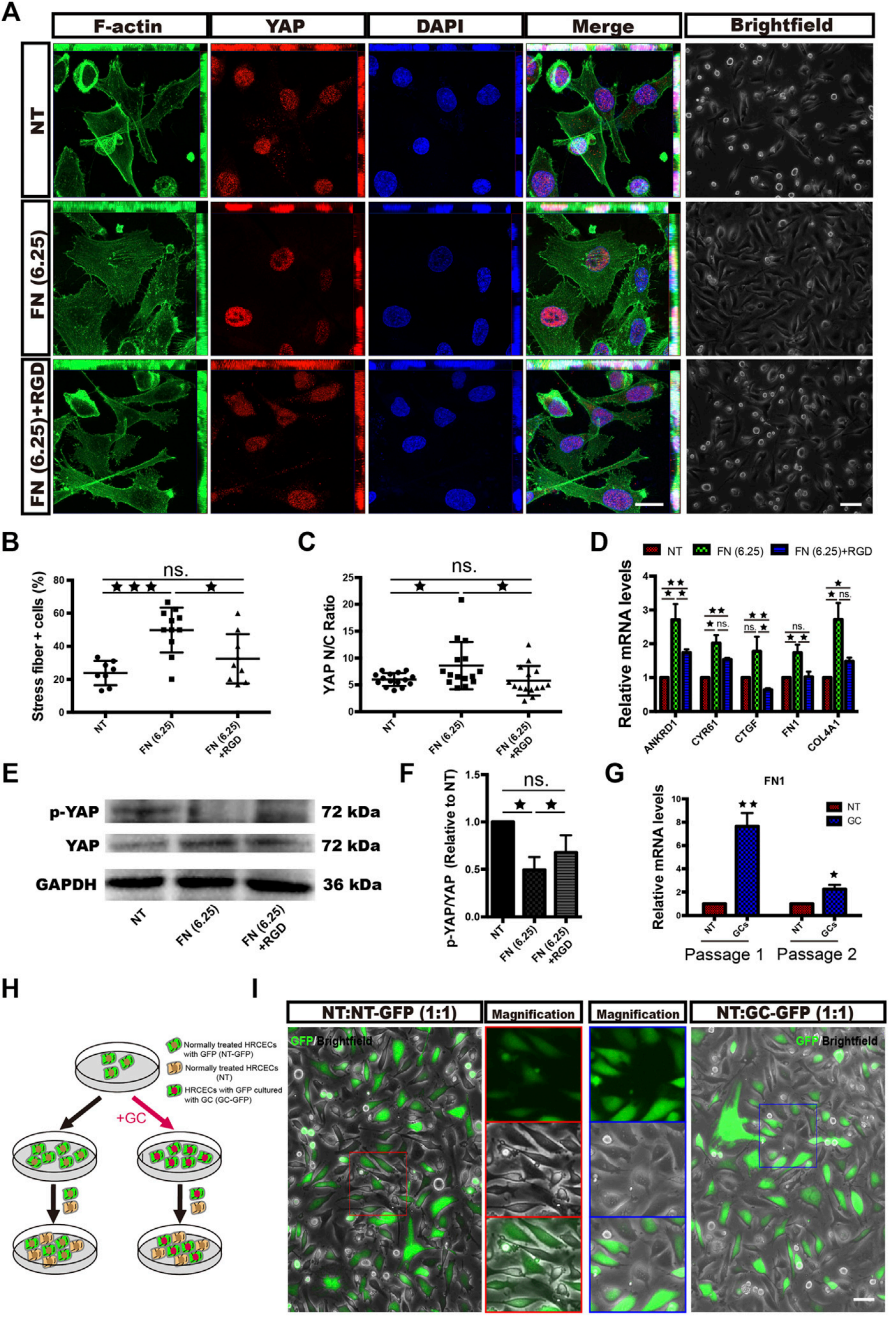
FN partially promotes stress fiber formation, YAP nuclear translocation and morphological changes in HRCECs via ECM-integrin signaling. **(A)**: F-actin/YAP/DAPI staining was performed by immunofluorescence in HRCECs cultured in RPMI 1640 Medium (Gibco) containing 10% FBS (Gibco) alone or in combination with 1 mg/ml RGD on dishes coated with 6.25 μg/cm^2^ FN for 72 h. Scale bars, 20 μm. Brightfield microscopy images of HRCECs treated under the indicated condition. Scale bars, 100 μm. **(B)**: Quantification of the percentage of cells displaying actin stress fibers for the three treatment conditions in **(A)**. **(C)**: Quantification of the ratio of the average immunofluorescence intensity of YAP between the nucleus and the cytoplasm of the cells in **(A)**. **(D)**: Real-time qPCR analysis of HRCECs treated as in **(A)**. The mRNA levels of YAP1 and ANKRD1/CYR61/CTGF (targets of YAP) were calculated. **(E)**: WB analysis of YAP and p-YAP of HRCECs treated as in **(A)**. p-YAP is phospho-S127. **(F)**: The p-YAP/YAP in **(E)** is quantified and normalized to NT group. **(G)**: Real-time qPCR analysis of passage-1 and passage-2 HRCECs. Passage-1 HRCECs were cultured in RPMI 1640 medium (Gibco) containing 10% FBS (Gibco) alone (NT) or in combination with 0.8 μM hydrocortisone (GC) for 72 h. Passage-2 HRCECs were derived from passage-1 cells. **(H)**: Flow chart of cell coculture. HRCECs with GFP were divided into two groups: one group was pretreated with hydrocortisone and cocultured with normal HRCECs at a ratio of 1:1 [the NT:GC-GFP (1:1) group], and the other was directly cocultured with normal cells at a ratio of 1:1 [the NT:NT-GFP (1:1) group]. **(I)**: Representative GFP fluorescence and brightfield images. Scale bars, 100 μm. The error bars represent the mean ± SD, and the experiment was biologically repeated three times. *p < 0.05; **p < 0.01; ***p < 0.001, ns., not significant. Student’s two-tailed paired t-test **(D,F,G)** or Student’s two-tailed unpaired t-test **(B,C)**. GFP green fluorescent protein, RGD Arg-Gly-Asp.

Given that ECM overexpression is a major effect after GC administration in HRCECs and that YAP mediated this event, we investigated whether the overexpressed ECM can activate YAP in turn. In the supernatant of HRCECs under GC treatment, we found only FN significantly increased, while the secretion of Col IV did not change (Figure 1F). So we conducted a series of experiments to define the role of the FN in YAP activation. First, we seeded cells on dishes coated with FN at a 6.25 μg/cm^2^ concentration (FN (6.25) group). Of note, cells treated with FN exhibited characteristics similar to those of cells treated with the GC. These characteristics included stress fiber formation, YAP nuclear translocation and ECM expression. Treatment of the cells with FN in combination with a synthetic peptide [the RGD-containing peptide; FN (6.25) + RGD group] that competes with ECM to bind with integrin reversed these changes to a certain extent, although the reversal was not complete (Figures 5A–F).

We also treated cells with Col IV at different concentrations and found that Col IV could not stimulate cells to change as FN did (Supplementary Figures 2A,B).

Next, HRCECs were treated with the GC alone or in combination with RGD. Unsurprisingly, RGD cotreatment prevented the YAP nuclear translocation, stress fiber formation, ECM expression and FN secretion induced by GC treatment (Supplementary Figures 2C-E).

The FN1 mRNA expression of passage-2 GC-pretreated HRCECs without extra GC supplementation was significantly higher than that of passage-2 NT HRCECs, although the elevated level was not as high as that of passage-1 cells (Figure 5G). We divided HRCECs with GFP into two groups: one group was cultured with the GC (passage 1) for 72 h, passaged (passage 2) and cocultured with NT HRCECs at a ratio of 1:1 [the NT:GC-GFP (1:1) group], and the other was directly cocultured with NT cells at a ratio of 1:1 [the NT:NT-GFP (1:1) group] (Figure 5H). Light and fluorescence microscopy analyses showed that the NT HRCECs in the NT:GC-GFP (1:1) group, but not those in the NT:NT-GFP (1:1) group, exhibited increases in cell size and spreading, and the cell edges became blurred instead of sharp (Figure 5I). This result indicated that the excessive FN secretion by the pretreated cells was able to induce YAP activation in adjacent normal cells.

### YAP is the Key Molecule in the Responsiveness of HRCECs to GC Treatment

**FIGURE 6:**
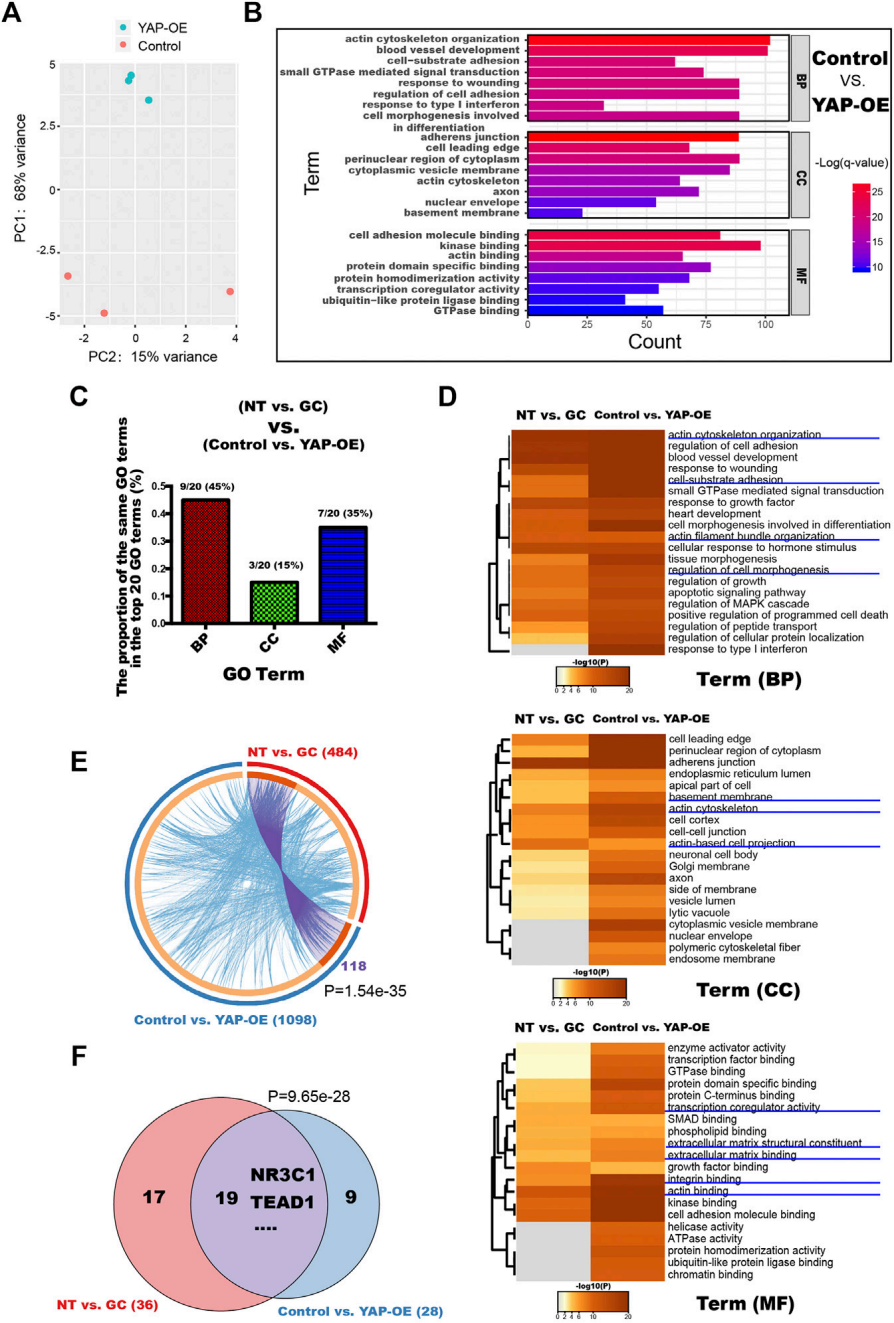
YAP is a key molecule related to ECM component expression and morphological changes induced by GCs in HRCECs. **(A)**: PCA was performed on samples from the YAP-OE vs. control comparison. HRCECs were cultured in RPMI 1640 medium (Gibco) containing 10% FBS (Gibco) for 72 h and then transfected with YAP-OE or control lentiviral vectors for 48 h. **(B)**: Top significantly enriched GO terms for the DEGs between the control group and YAP-OE group for the BP, MF and CC categories. **(C)**: The proportion of the same GO terms in the top 20 significantly enriched GO terms between the NT vs. GC groups and control vs. YAP-OE groups for the BP, MF and CC categories. **(D)**: Dendrogram of the enriched GO BP, CC and MF terms across the DEGs from the NT vs. GC and YAP-OE vs. control comparisons. The heatmap cells are colored by their *p* values. **(E)**: Gene overlap analysis. The Circos plot shows how the DEGs from the NT vs. GC comparison and the YAP-OE vs. control comparison overlap. Purple lines link the same gene that is shared by multiple gene lists. Blue lines link different genes associated with the same ontology term. A greater number of purple links and a longer dark orange arc imply greater overlap among the input gene lists. The blue links indicate the amount of functional overlap among the input gene lists. **(F)**: Venn diagrams showing the TF number overlap in the TF binding site enrichment analysis of DEGs from the NT vs. GC and YAP-OE vs. control comparisons. The analysis was performed with a web-accessible software program called oPOSSUM-3. In **(E) and (F)**, *p* indicates the *p*-value of the overlap in Venn diagrams and Circos plot from Fisher’s exact test. DEGs differentially expressed genes, GO Gene Ontology, BP biological process, CC cellular component, MF molecular function, TF transcription factor.

To gain further insight into the role of YAP in the responsiveness of HRCECs to GCs, we transfected HRCECs with YAP-OE or control lentiviral vectors and then used mRNA sequencing to analyze the impact of YAP on the whole gene expression profile of HRCECs. PCA showed that samples from the YAP-OE group could be separated from samples from the control group (Figure 6A). GO analyses were performed with a set of 1098 DEGs between the YAP-OE and control groups (Figure 6B). Of note, a considerable proportion of the terms that were among the top significantly enriched GO terms was similar to those for the NT vs. GC comparison, including blood vessel development, regulation of cell adhesion, extracellular matrix organization, actin binding and others (Figures 2B, 6B,C). We analyzed all significantly enriched GO terms across the NT vs. GC and YAP-OE vs. control comparisons and found that additional terms were shared between the two analyses (Figure 6D). Aside from the aforementioned terms, other common terms included actin cytoskeleton organization, cell-substrate adhesion, regulation of cell morphogenesis, BM, transcription coregulator activity, integrin binding, etc. (Figure 6D). The abovementioned same GO terms in the two analyses have been verified to a certain extent in our experiments.

Moreover, we compared the 1098 DEGs from the YAP-OE vs. control comparison with the 484 DEGs from the NT vs. GC comparison. Although 118 identical genes (linked with purple lines) were significantly shared by the two gene lists, many different genes (linked with blue lines) were associated with the same ontology terms, indicating the existence of a large amount of functional overlap between the input gene lists (Figure 6E).

According to the TF prediction analysis of the two RNA-seq datasets, significant overlap was observed between enriched TFs, confirming that common transcriptional networks are present in the two datasets (Figure 6F). In addition, we found that NR3C1 and TEAD1 were enriched in both datasets.

Given the similarity of GO terms, DEGs and TFs between the NT vs. GC and YAP-OE vs. control comparisons, it is reasonable to speculate that YAP is the key molecule in the responsiveness of HRCECs to GC treatment.

**FIGURE 7:**
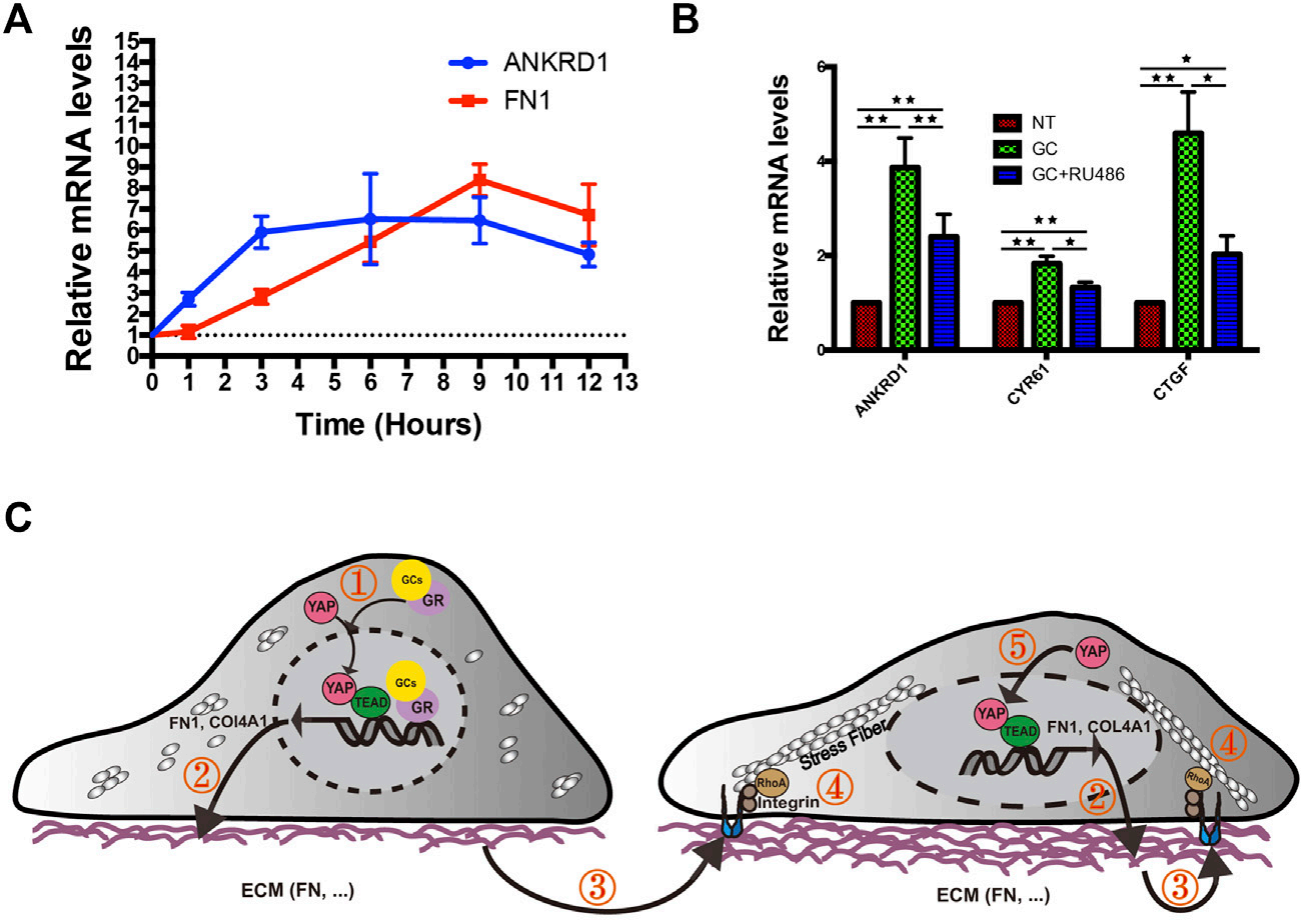
Proposed working model summarizing the results from our study. **(A)**: Real-time qPCR analysis of HRCECs treated with 0.8 μM hydrocortisone for the indicated times. The mRNA levels of FN1 and ANKRD1 (targets of YAP) were calculated. The experiment was biologically repeated three times. **(B)**: Real-time qPCR analysis of HRCECs treated with 0.8 μM hydrocortisone alone or in combination with 10 μM RU486 for 72 h. The mRNA levels of ANKRD1/CYR61/CTGF (targets of YAP) were calculated. The experiment was biologically repeated four times. **(C):** Schematic diagram showing the transcriptional positive feedback loop that regulates ECM component expression in GC-treated HRCECs. GC promotes YAP nuclear localization to induce ECM component (FN and Col IV) expression (① and ②). The increase in FN acts on integrin to promote stress fiber formation (③ and ④), which applies forces to the nucleus and flattens it. This change in turn leads to increased YAP import through the stretched nuclear pores (⑤). The error bars represent the mean ± SD. *P< 0.05, **P< 0.01. Student’s two-tailed paired *t*-test **(B)**. ECM extracellular matrix, FN fibronectin.

Since we have previously confirmed that YAP and FN can regulate each other, we wanted to explore which is affected first in HRCECs under GC treatment. We monitored the time course of FN expression and YAP activation after GC treatment using the mRNA levels of FN1 and YAP target genes (ANKRD1) as readouts. The results showed that YAP activation preceded FN expression upon GC treatment (Figure 7A), indicating that the underlying mechanism that initially promotes YAP activation needs to be further explored. RU486 can inhibit GR nuclear translocation. Our results also showed RU486 can inhibit YAP activity (Figure 7B), so GR may mediate the initial YAP activation.

Upon engagement with ECM, integrin can active signaling such as Rho GTPases family (Heng and Koh, 2010), which induce the formation of dynamic actin-containing structures such as lamellipodia, filopodia, and stress fibers (Heasman and Ridley, 2008). Among the Rho GTPases family, at least RhoA directly promotes stress fiber assembly through its effectors (Leung et al., 1996; Watanabe et al., 1997).

Based on these results and published literatures, we created a diagram of a plausible mechanism. Upon GC stimulation, FN expression in HRCECs is increased by YAP nuclear localization and activation. Cells interact mechanically with overexpressed FN via integrins, which promote stress fiber formation mediated by RhoA. In turn, stress fibers apply forces to the nucleus, flattening it, stretching nuclear pores, and eventually resulting in an increase in YAP nuclear import. Upon YAP nuclear entry increase, the above pathways will be activated again, thus forming a positive feedback loop (Figure 7C).

### YAP Activation in Vascular ECs of Diabetic Patients and STZ-Induced Diabetic Mice

**FIGURE 8:**
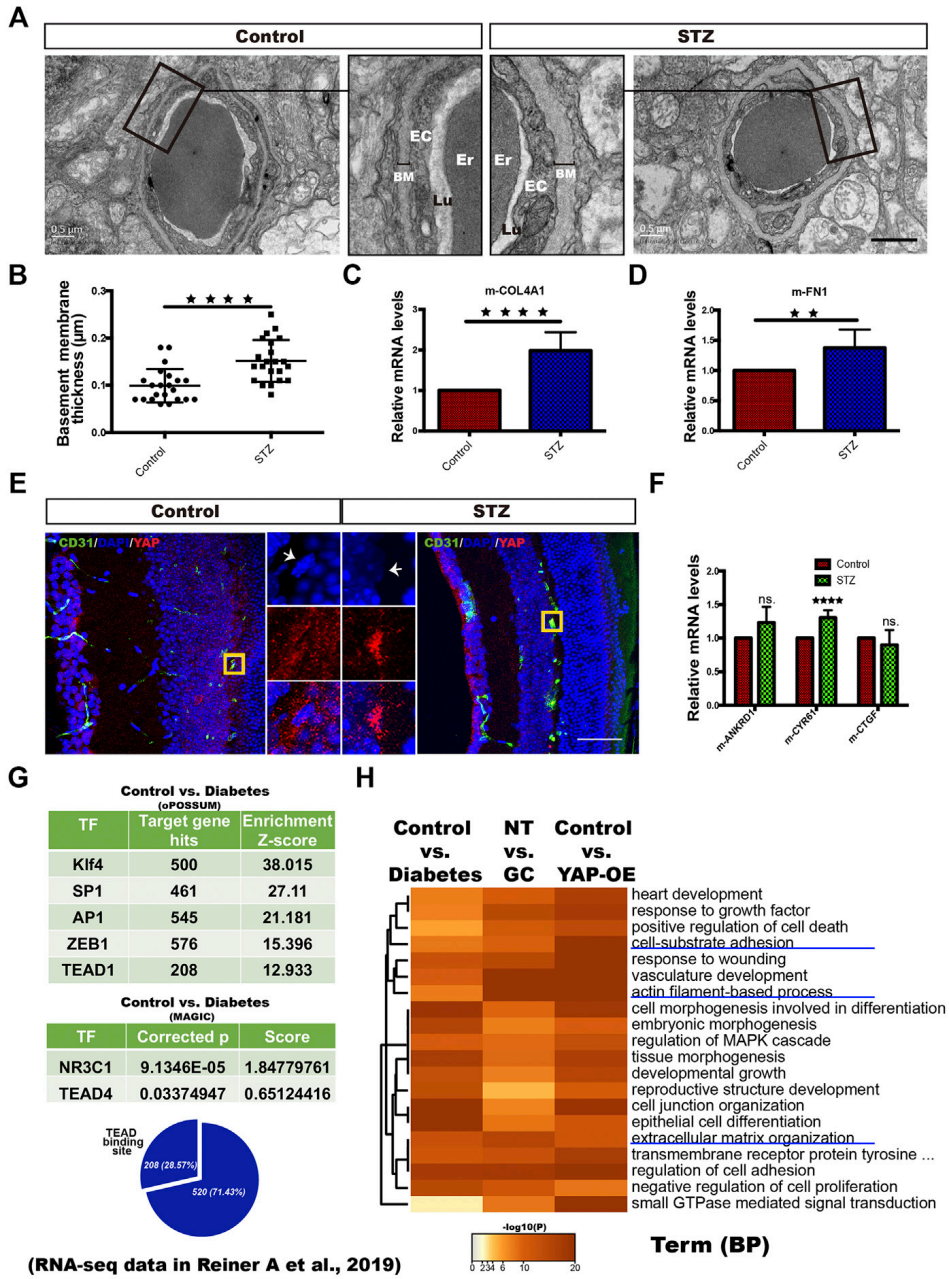
YAP activation in vascular ECs of diabetic patients and STZ-induced diabetic mice. **(A)**: TEM images of retinal capillaries of STZ-induced diabetic mice and control mice. The magnified views show thickening of the BM in STZ-induced diabetic mice. EC endothelial cell, Er erythrocyte, Lu lumen. Scale bars, 1 μm. **(B)**: Basement membrane thickness of retinal capillaries of STZ-induced diabetic mice and control mice. **(C and D)**: Real-time qPCR analysis of retinal tissue from the control and STZ-induced diabetic mice. The mRNA levels of FN1/COL4A1 (ECM components) were calculated. **(E)**: CD31/DAPI/YAP immunofluorescence staining of retinal sections from the control and STZ-induced diabetic mice. Scale bars, 50 μm. **(F)**: Real-time qPCR analysis of retinal tissue from the control and STZ-induced diabetic mice. The mRNA levels of ANKRD1/CYR61/CTGF (targets of YAP) were calculated. **(G)**: Selected factors found in the TF binding site enrichment analysis of DEGs from the control vs. diabetes comparisons. The analysis was performed through two methods. oPOSSUM-3 is a web-accessible software program. The MAGIC algorithm was used for the analysis in Python. The proportion of significantly differentially regulated genes in the DEGs with predicted TEAD binding sites in promoter regions. Samples from four patients with T2DM (diabetes) and six nondiabetic controls (control) were included in the analysis based on the RNA-seq data of dermal vascular ECs in the published literature (Reiner A et al., 2019; GEO accession number: GSE92724). **(H)**: Dendrogram of the enriched GO BP terms across the significant DEGs from the diabetes vs. control, NT vs. GC and YAP-OE vs. control comparisons. The heatmap cells are colored by their *p* values. The error bars represent the mean ± SD **(B,C,D,E)**. The experiment was biologically repeated three times **(A and E)**, nine times **(C and D)** and six times **(F)**. **p < 0.01; ****p < 0.0001; ns., not significant Student’s two-tailed paired t-test **(C,D,F)** or Student’s two-tailed unpaired t-test **(B)**. STZ streptozotocin, GO Gene Ontology, TF transcription factor, DEGs differentially expressed genes, BP biological process.

To test whether YAP is active in ECs in the context of DM *in vivo*, we established a DM mouse model with streptozotocin (STZ) as previously reported (Rong et al., 2018). This mouse model was also reported to have elevated corticosterone levels (Erickson et al., 2017). After 3 months of constant hyperglycemia, the mice were sacrificed, and the retinal microvasculature was assessed. TEM analysis revealed that the thickness of the blood vessel BM was obviously increased in diabetic mice compared with nondiabetic mice (Figures 8A,B). In addition, the mRNA expression of COl4A1 and FN1 was remarkably increased in the retinal tissue of the diabetic mice (Figures 8C,D). Next, immunofluorescence staining demonstrated YAP nuclear translocation in the ECs of the deep blood plexus in the diabetic mouse retina (Figure 8E). Moreover, CYR61 mRNA expression was significantly higher in the retinas of the diabetic mice than in those of the control mice (Figure 8F). These results suggest that YAP is activated in ECs of the STZ-induced diabetic mice.

Human patients with untreated DM always have elevated plasma cortisol levels (Erickson et al., 2017). Through an in silico approach, we reanalyzed RNA-seq data of dermal vascular ECs from four patients with T2DM and six nondiabetic controls in the published literature. PCA showed distinct clustering of diabetic patient-derived ECs compared with ECs from nondiabetic controls (Supplementary Figure 3A). TF prediction analysis showed that NR3C1, TEAD and TFs that cooperate with YAP-TEAD, such as Klf4, SP1, AP1, MYC and ZEB1, were enriched in the DEGs of the diabetes vs. control comparison (Figure 8G, Supplementary Figure 3B). Next, GO analysis revealed that the DEGs across the diabetes vs. control, NT vs. GC and YAP-OE vs. control comparisons were enriched for many of the same BP terms, including cell-substrate adhesion, actin filament-based process, and extracellular structure organization (Figure 8H). These results suggest that GR and YAP are activated in ECs of T2DM patients.

## Discussion

GCs are vital steroid hormones involved in the regulation of body composition, metabolism, neurocognition, neuropsychiatric function and the immune system in response to a variety of environmental and physiological stimuli (De Leo et al., 2010; Del Rincón et al., 2014; Geer, 2016). T2DM can activate the hypothalamic-pituitary-adrenal (HPA) axis, which leads to continued hypersecretion of cortisol, and the presence of chronic complications further increases HPA hyperactivity (Prpić-Križevac et al., 2012). Increases in GC levels (even subtle increases) have been demonstrated to worsen diabetes, as reflected by deterioration of metabolic control and increases in the occurrence of complications, such as DR (Chiodini et al., 2007; Geer, 2016). Because of the plausible role of the retinal endothelium in the pathophysiology of DR, a plethora of *in vivo* studies have used isolated retinal capillary endothelia to assess molecular and cellular responses to factors associated with diabetes (high glucose, lipids, shear stress, cytokines, growth factors, etc.), and such studies have provided valuable information regarding the pathogenic mechanisms in these cells (Stitt et al., 2016). To verify the hypothesis that a direct link exists between GCs and DR, we conducted the present study using HRCECs and demonstrated that GC stimulation can induce ECM component (FN and Col IV) expression and morphological changes in HRCECs in a manner dependent on YAP activation.

BM thickening of capillaries, the major and earliest structural alteration of diabetic microangiopathy, occurs not only in the retinas but also in the kidneys, muscle and skin in diabetic patients and animal models (Bianchi et al., 2016). The BM is composed of several highly structured components, including Col IV, FN and laminin, which are produced primarily by ECs and pericytes (Roy et al., 2010). In addition to hyperglycemia, increased growth factor activity, protein kinase C (PKC) activation, advanced glycation end-product (AGE) accumulation, inflammation and other factors are also associated with BM thickening, although the mechanisms of action of these factors remain uncertain (Roy et al., 2010). Our results identify GCs as new candidate factors for BM thickening in DR and provide a related molecular mechanism, elucidating a scenario in which excess GCs may affect the initiation and progression of DR.

Systemic GC excess, caused by either Cushing’s syndrome or chronic stress, is associated with some CVDs, such as systemic atherosclerosis, and a large body of evidence supports GC-mediated endothelial dysfunction as a key mechanism in this disease (De Leo et al., 2010; Del Rincón et al., 2014; Geer, 2016; Sher et al., 2020). Atherosclerosis is a disease of large and medium-sized arteries and is the most frequent underlying cause of coronary artery disease, carotid artery disease, and peripheral arterial disease (Falk, 2006). GC-induced endothelial dysfunction of large and medium-sized arteries involves primarily decreased endothelial nitric oxide (NO) synthesis and increased expression of adhesion molecules, which contribute to the progression of inflammation (Sher et al., 2020). Interestingly, we observed that few inflammation-related pathways were enriched in our RNA-seq study. However, our results are consistent with some observations from experiments in which ECs from the microvasculature were treated with GCs. Regarding brain capillary ECs, some studies have found that GCs promote morphological changes and gene or protein expression changes, including F-actin rearrangement to form “fiber-like” structures, rearrangement of VE-cadherin protein in the cytoskeleton and increased expression of Col IV a1 and the laminin a5 subunit, although the precise mechanisms of these effects remain unclear (Weidenfeller et al., 2005; Blecharz et al., 2008; Thomsen et al., 2017). There have also been some inconsistent observations regarding cells in the human skin microcirculation, bovine endothelial retinal cells, and flat-mounted rat retinas. The degree of GC-induced EC death varies according to the EC type and the chemical properties of the GC (El Zaoui et al., 2015). In our study, we cultured HRCECs with 0.8 μM hydrocortisone, which is beyond the physiological upper range of serum cortisol values, and found not only that it did not promote apoptosis but also that it promoted cell proliferation and migration. Combined, our observations and those in previous studies indicate that EC heterogeneity in organs and blood vessel types plays an important role in the response to GCs and support the use of HRCECs to study how GCs affect DR.

The diabetes-associated chronic low-grade inflammation is accepted to be an important part of the pathogenesis of DR (Yellowlees Douglas et al., 2012; Chakravarthy et al., 2016). It suggests that the control of inflammation may be an effective way to treat DR, which is evidenced by the clinical efficacy of intravitreal corticosteroid agents in diabetic macular edema (DME) (Boyer et al., 2014; Daruich et al., 2015a). GCs can mediate anti-inflammatory or pro-inflammatory activities depending on the context (Desmet and De Bosscher, 2017). In DME, local inflammation is a major and permanent pathogenic mechanism, GCs promote the regression of edema through anti-inflammatory effects (Daruich et al., 2018). DME is a complication of advanced diabetes, while the pathogenesis of early diabetic retinopathy was explored in this study. In the early diabetic retinopathy, we speculate GCs may play a role, like extracellular matrix remodeling, instead of anti-inflammatory, due to the context of different stages of DR is different (Stitt et al., 2016).

Our experiment found a positive feedback loop in GC-treated HRCECs in which YAP nuclear translocation promoted ECM component (FN and Col IV) expression. Increases in the levels of FN and Col IV, especially FN, enhanced ECM stiffness to sustain YAP activity. Similar positive feedback loops mediated by YAP with different molecular mechanisms have also been reported in breast cancer cells (Chang et al., 2015), breast cancer-associated fibroblasts (CAFs) (Grande-Garcia et al., 2013), mechanically activated mesenchymal stem cells (MSCs) (Nardone et al., 2017) and other cell types. In ECs, during vascular development, VEGF induces cytoskeletal rearrangement to activate YAP, which feeds back to cytoskeletal gene expression to sustain endothelial remodeling (Kim et al., 2017). Our study illustrates a new feedback mechanism in ECs and indicates that YAP impacts a variety of EC events depending on the cell subtype, the specific pathological or physiological state and the responsiveness to growth factors and hormones.

F-actin cytoskeleton organization is now broadly accepted as a dominant input regulating YAP subcellular localization and activity, but the exact mechanisms are elusive (Totaro et al., 2018). Two main hypothetical scenarios have been proposed. In one scenario, YAP normally binds with inhibitory proteins to remain inactive, and F-actin interacts with these inhibitory proteins in a manner mutually exclusive with their YAP association (Zhao et al., 2011; Mana-Capelli et al., 2014). In the second scenario, stress fiber formation allows the establishment of a mechanical connection between the nucleus and the cytoskeleton and applies cytoskeletal tension to the nucleus, which leads to nuclear flattening, permeability of nuclear pores and an increase in YAP nuclear entry (Elosegui-Artola et al., 2017). In the present study, we inhibited F-actin stress fiber formation and retained YAP in the cytoplasm and found that stress fiber formation increased the size of the nucleus; these findings implied that the second F-actin-mediated YAP nuclear entry mechanism was functional in our study. Unfortunately, due to technical limitations, we did not further verify this pathway in HRCECs.

In this study, the actions of the GC on HRCECs were mediated by GR, a member of the nuclear receptor family encoded by NR3C1 and a ligand-dependent TF (Desmet and De Bosscher, 2017). The details of the GR signaling-dependent YAP pathway were previous reported in breast cancer cells, where GR signaling activates YAP through FN-mediated integrin activation and F-actin polymerization (Sorrentino et al., 2017). Although increases in FN expression can activate YAP, we found that YAP activation preceded FN expression in HRCECs under GC stimulation, so the mechanism by which GCs initially activate YAP before FN expression remains unclear. Based on the fact that RU486 can prevent YAP activation induced by GCs and the finding that NR3C1 was enriched in our TF prediction analysis performed with the YAP-OE RNA-seq data, we hypothesize that GR nuclear translocation may participate in YAP nuclear translocation via direct or indirect protein-protein interactions. However, *in situ* proximity ligation assays (PLAs) showed no apparent interaction of YAP with GR (data not shown). Additionally, upon binding to GC-responsive elements (GREs), GR can directly recruit cofactors, such as TIF2 and SRC-1, through mechanisms that have not been fully defined (Lonard and O’Malley, 2005). In view of the complexity of the mechanism, we will investigate the exact mechanism in a future study for practical reasons.

In the STZ-induced diabetic mice, the elevated cortisol levels are consistent with those of human patients with untreated DM (Erickson et al., 2017). Moreover, in the retinas of the STZ-induced diabetic mice and in the dermal skin of patients with T2DM, BM thickening is a main hallmark of diabetic vasculopathy (Kern et al., 2010; Wimmer et al., 2019). YAP exhibited nuclear accumulation in retinal ECs of the STZ-induced diabetic mice. Bioinformatics analysis also indicated that GR and YAP were activated in the ECs of the dermal skin microvasculature of T2DM patients. These *in vivo* findings corroborate the results of our *in vitro* experiments and inspire us to further verify the role of YAP activation in BM thickening and the relationship between GCs and YAP *in vivo*.

It is becoming increasingly apparent that DR imposes huge social and economic burdens (Leasher et al., 2016); in addition, the current treatments for DR are directed toward advanced stages and are often destructive (Duh et al., 2017). Thus, research on the early pathogenesis of DR warrants attention, and treatments that are preventative or address early pathology are highly desirable. The data presented in this work suggest that hypersecretion of GCs may play critical roles in DR initiation and progression and depict the mechanism by which GCs lead to ECM component expression in a YAP-dependent manner in HRCECs. The findings also indicate that the GC-YAP-ECM axis may be a new target for inhibiting vascular BM thickening during the early pathogenesis of DR.

## Data Availability

All datasets generated for this study are included in the article/Supplementary Material, further inquiries can be directed to the corresponding author/s. The RNA-seq data present in the study are deposited in the SRA repository, accession numbers PRJNA746680 and PRJNA750725.
