# Supplementary figures and images for: Glucocorticoids Promote Extracellular Matrix Component Remodeling by Activating YAP in Human Retinal Capillary Endothelial Cells
Source: Front Cell Dev Biol. 2021 Dec 14;9:738341. doi: 10.3389/fcell.2021.738341 (PMC8712730; doi:10.3389/fcell.2021.738341)

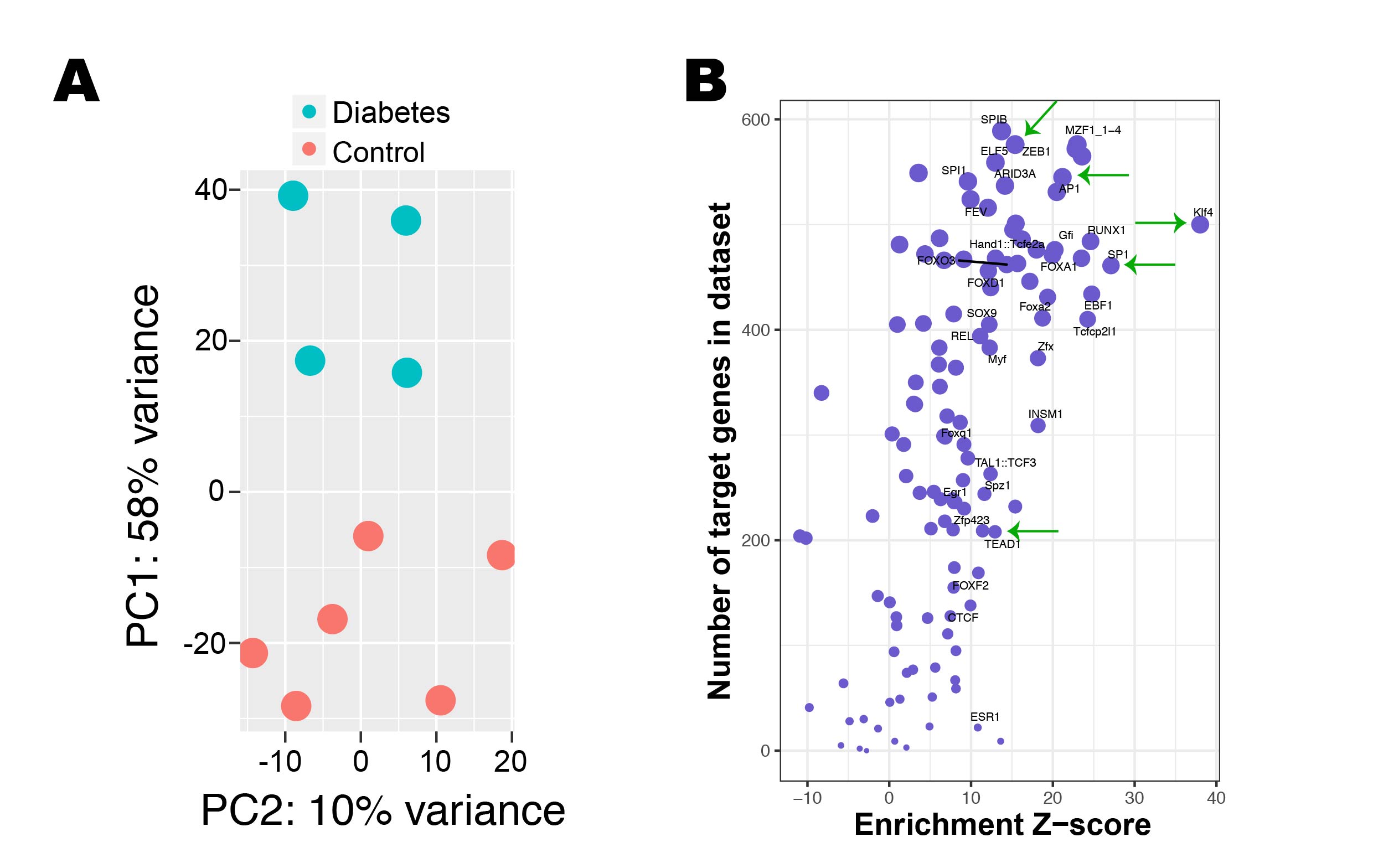

Supplement: Supplementary file 2 [file Image3.JPEG]

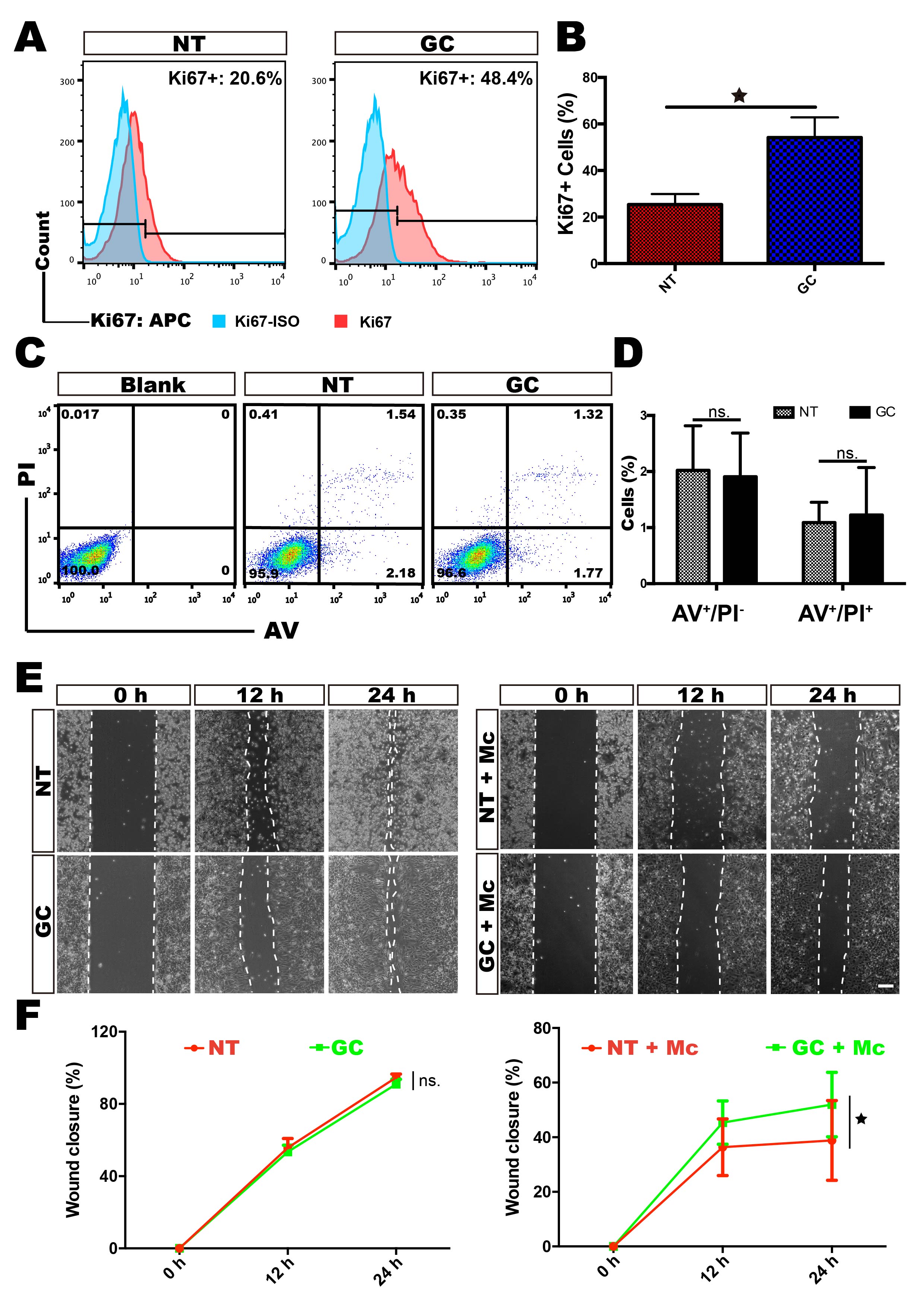

Supplement: Supplementary file 3 [file Image1.JPEG]

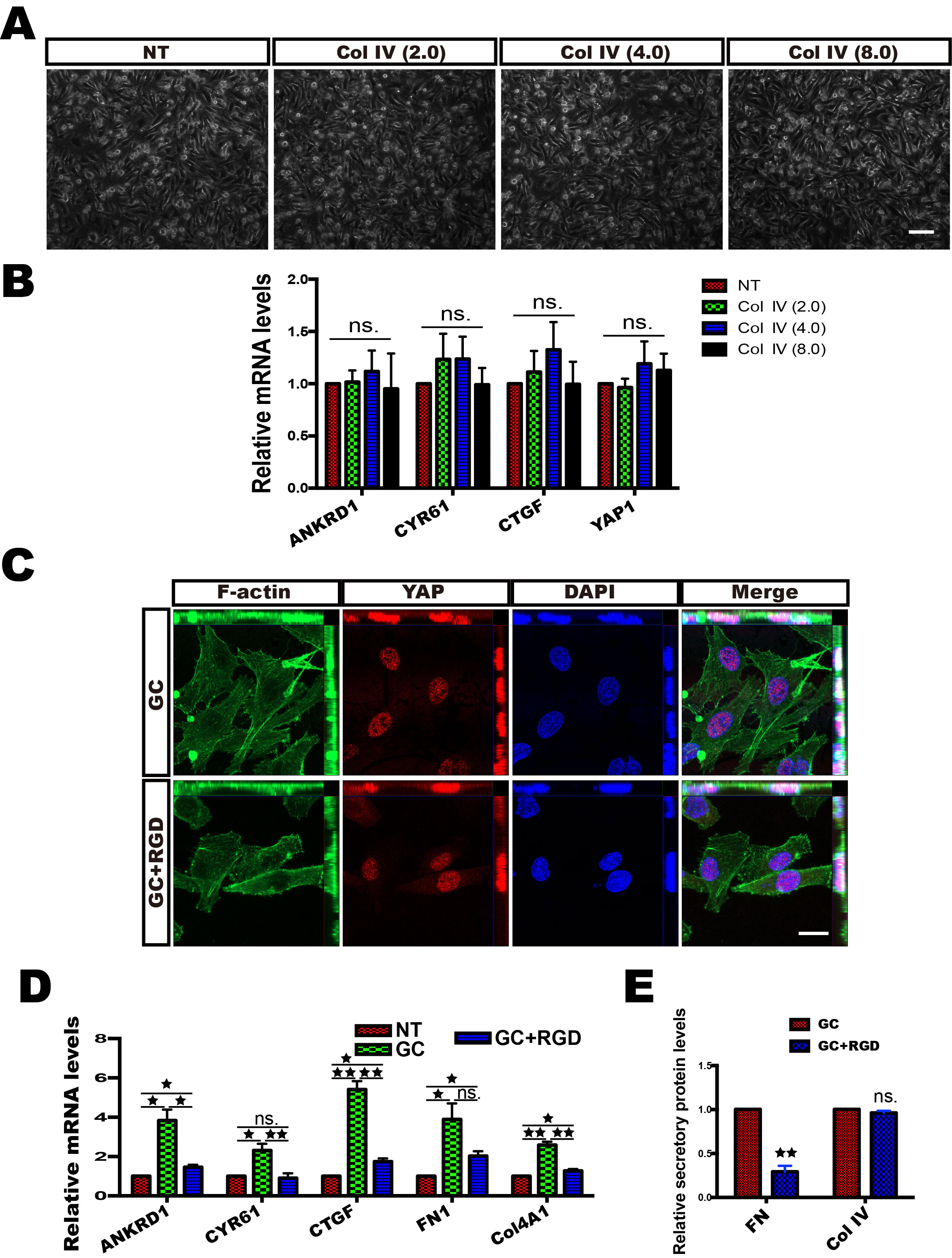

Supplement: Supplementary file 4 [file Image2.JPEG]
